# The mediating role of emotional exhaustion in the relationship between normative commitment and pay satisfaction among university counselors

**DOI:** 10.3389/fpsyg.2026.1810414

**Published:** 2026-04-29

**Authors:** Zeng Cheng, Esayas Teshome Taddese, Bereket Merkine Gebresilase, Mitiku Tasisa Dinsa, Kelemu Zelalem Berhanu, Melaku Takele Abate, A. Shorouk

**Affiliations:** 1 Faculty of Education and Liberal Arts, INTI International University, Nilai, Malaysia; 2 College of Physics and Electronic Information Engineering, Minjiang University, Fuzhou, China; 3 School of Nursing, Shandong Xiehe University, Jinan, China; 4 Department of English Language and Literature, College of Social Science and Humanities, Wolkite University, Welkite, Ethiopia; 5 Department of Education Leadership and Management, University of Johannesburg, Johannesburg, South Africa; 6 Jimma College of Teachers’ Education, Jimma, Ethiopia; 7 Department of Education, College of Humanities and Science, Ajman University, Ajman, United Arab Emirates

**Keywords:** education, educational governance, emotional exhaustion, normative commitment, pay satisfaction

## Abstract

This study examines the association between normative commitment and pay satisfaction among university counselors moderated by emotional exhaustion. The study employed quantitative, cross-sectional design, involving a sample of 294 university counselors working at three universities in China. The results confirmed that higher normative commitment is related to both lower emotional exhaustion and higher pay satisfaction. It also proved that emotional exhaustion mediates the association between normative commitment and pay satisfaction. This shows that the counselors’ perceived moral obligation toward their institution contributes to job satisfaction. This research contributes to the understanding of organizational commitment and its impact on work outcomes in higher education, offering practical implications for improving counselor retention and satisfaction through targeted support and compensation systems.

## Background

Over the past decades, there has been continuous increase in student enrollment China’s higher education system. In connection with this, universities face increasingly complex governance challenges, particularly in the areas of student management, ideological guidance, and service delivery (Lei et al., 2025). This situation has significantly increased the workload of the university counselors. The counselors in Chinese universities are the key link between the governance of institutions and the students. They are involved in psychological counseling, daily management of student cases, responding to crises and provision of developmental guidance. Besides, they have key roles handling ideological and political education students at universities. This multifaceted role demands sustained emotional engagement, strong moral responsibility, and professional dedication (Liang and Yin, 2025). Motivation for staying in this role is heavily tied to a sense of moral responsibility and institutional loyalty, which can be seen as an indicator of high normative commitment. As the number of students continues to rise, the workload of university counselors has also continued increasing, leading to increased stress. Long-term exposure to such intense emotional labor, frequent interpersonal interaction, and consistent demands for responsiveness can result in the exhaustion of counselors’ emotional resources. The consequences include increased susceptibility to emotional exhaustion that compromises their ability to engage with their work and maintain their psychological well-being (Guan et al., 2025; Ding et al., 2022).

Although normative commitment can motivate counselors to endure increased job demands, excessive emotional exhaustion weakens the positive impact of such commitment. Previous studies theorized that emotionally exhausted employees are more likely to have less positive evaluation of their work conditions, even when their pay is fair (Gebresilase et al., 2025; Tsang et al., 2022). Pay satisfaction is an essential factor affecting employees’ retention intentions, yet prolonged feelings of emotional exhaustion may lead to reduced satisfaction, even if salary levels remain objectively consistent. In Chinese context, many university counselors experience minimal salary growth, and an inadequate alignment between their workload and compensation levels (Wang et al., 2021). Empirical research suggests that lower levels of pay satisfaction may contribute to reduced occupational well-being (Amoozegar et al., 2025; Wang et al., 2021; Han and Wang, 2025).

It is therefore important to better understand the process through which normative commitment translates into pay satisfaction. Such research can assist in better understanding counselors’ work psychology and provide empirical evidence to improve the remuneration system, mitigating burnout, and support sustainable development of higher education governance (Wei and Ye, 2022; Han and Wang, 2025).

In the field of organizational psychology, a lot of studies have found that organizational commitment can affect pay satisfaction and that emotional exhaustion is a critical mediator (Wang et al., 2020; Zhang et al., 2021). However, there has been limited studies on this particular field focused on Chinese higher education context and, more specifically, the role of university counselors. In China, university counselors serve both as educator and administrative officer, which exposes them to a high level of job pressure and emotional labor. The dual nature of their role makes university counselors particularly prone to emotional exhaustion due to the high workload and emotions involved in their job. However, so far, only a small number of empirical studies have been conducted targeting this particular group (Gazi et al., 2025; Zavaleta et al., 2025).

Furthermore, whereas Meyer and Allen (1991) introduce three dimensions of organizational commitment, namely- affective commitment, continuance commitment, and normative commitment, few studies have explored how these dimensions can independently affect emotional exhaustion and pay satisfaction. This narrow approach impedes a comprehensive understanding of the underlying mechanisms and limits the formulation of targeted interventions.

This study aims to investigate the impact of normative commitment on pay satisfaction for counselors at three Chinese universities, with emotional exhaustion serving as a mediating variable. Earlier studies suggest that the relationship between normative commitment, pay satisfaction, and emotional exhaustion is context-dependent (Torrelles-Nadal and Chang, 2025; Costa Dutra et al., 2025). By parsing these relationships within the specific context of Chinese university counselors, this research seeks to gain a clearer understanding of how normative commitment affects pay satisfaction and whether emotional exhaustion mediates this process. This study not only contributes to the body of knowledge on human resource management in higher education frameworks but also provides pragmatic insights for designing interventions aimed at boosting counselors’ pay satisfaction, ultimately supporting universities in the modernization of governance systems. For this, the study aims to examine the relationships among normative commitment, emotional exhaustion, and pay satisfaction among university counselors, and to test whether emotional exhaustion mediates the association between normative commitment and pay satisfaction. The research questions that guided the study were:

1. What effect does normative commitment have on emotional exhaustion for university counselors?
2. What effect does normative commitment have on pay satisfaction for university counselors?
3. What effect does emotional exhaustion have on pay satisfaction for university counselors?
4. Does emotional exhaustion mediate the relationship between normative commitment and pay satisfaction for university counselors?

## Literature review

In accordance with Meyer and Allen (1991) three-component model of organizational commitment. Organizational commitment comprises affective, normative, and continuance components, corresponding to emotional attachment, moral obligation, and the perceived loss associated with quitting. Previous studies have consistently shown that affective commitment has a negative correlation with emotional exhaustion, as emotionally attached employees are more likely to be resilient to work-related stress and burnout (Taddese et al., 2025; Karakus et al., 2014). Furthermore, continuance commitment has often been linked to higher emotional exhaustion, because when employees are unable to quit due to financial reasons or lack of alternative employment, they may feel trapped and psychologically conflicted (Knudsen et al., 2006). However, empirical evidence on normative commitment is less consistent, with some studies suggesting a buffering effect of value internalization, while others have found a stronger emotional strain on obligation-based persistence under heavy workloads (Liang and Yin, 2025).

In addition to this, organizational commitment was shown to influence pay satisfaction—which is an important factor in job satisfaction. Employees who possess high levels of affective and normatively committed attitudes are likely to perceive compensation systems as equitable and acceptable despite working with fewer financial rewards (Ducharme et al., 2007). These individuals could also perceive pay policies more positively due to stronger identification with organizational purposes and values (Azizan et al., 2016).

More recently, some scholars have emphasized the significant role of emotional exhaustion as a mediating variable between organizational commitment and job satisfaction. Emotional exhaustion depletes employees’ psychological resources and compromises their ability to translate positive organizational attitudes into favorable appraisals of rewards, thereby weakening the link between commitment and compensation (Bynum, 2019).

Despite these contributions, existing literature has largely considered organizational commitment to be a unidimensional construct. In particular, while normative commitment can involve strong moral obligation, which can either buffer emotional exhaustion or exacerbate its adverse effects, these dynamics have yet to be thoroughly investigated, especially in the context of university counselors. Addressing this gap will be critical for the development of a more nuanced theoretical framework and for the design of targeted human resource interventions in higher education settings (Knudsen et al., 2006).

## Theoretical framework

This paper is motivated by the Job Demands-Resources (JD-R) model that claims that employee well-being and work outcomes will be affected by the balance between job demands and job resources (Demerouti et al., 2001; Han et al., 2020). The term job demands refers to characteristics of work that require continuous physical or psychological effort, often resulting in physiological or psychological strain. Conversely, job resources are features of work that help people to attain work goals, alleviate job demands, or pursue personal growth and development. The JD-R model posits that excessive job demands can cause strain and burnout, especially emotional exhaustion, and sufficient job resources can mitigate the adverse effects of demands and promote positive attitudes toward work (Bardhoshi and Um, 2021).

Counselors in universities work in environments characterized by high emotional demands, role overload, and constant interpersonal interaction with students. Because of these characteristics, emotional exhaustion has been regarded as a particularly salient consequence of chronic experience of job demands (Park et al., 2021). With that in mind, organizational commitment can be seen as a valuable psychological resource that shapes how counselors respond to job demands.

### Normative commitment

In this study, normative commitment is considered a key psychological resource that may influence how university counselors respond to demanding work conditions. Specifically, Normative commitment (moral obligation, to stay with the organization) serves as the key factor in the counselor’s reactions to the demands of their work (Arthur et al., 2025). While the JD-R framework highlights multiple dimensions of emotional regulation, normative commitment appears to play a distinct role. When employees feel a sense of moral responsibility to their organization, especially when this responsibility is grounded in internalization of values and perceived organizational goals as legitimate, normative commitment acts as a significant psychological resource (Manuti et al., 2022).

In emotionally intense work contexts, a sense of moral duty to an organization can provide employees with a higher quality of purpose and meaning, aiding in the cognitive reappraisal of job demands, and supporting emotional regulation. For university counselors, who typically operate within a service, responsible, and value alignment framework, normative commitment may help them achieve greater endurance and emotional balance by strengthening their sense of role identity and professional mission (Zhang et al., 2025). Hence, normative commitment is theorized to act as a buffer against the adverse impacts of high emotional demands and minimize emotional exhaustion in this context.

### Pay satisfaction

Another important variable in this framework is pay satisfaction, which reflects employees’ perceptions of organizational rewards and compensation systems. In addition to its impact on emotional exhaustion, normative commitment also influences counselors’ assessments of pay satisfaction. Pay satisfaction is an important attitudinal outcome that captures employees’ perceptions of fairness and adequacy of organizational rewards. In the JD-R framework, normative commitment can bolster positive perceptions of reward systems by enhancing trust, identification, and acceptance of organizational policies, even in the presence of restricted compensation (Sarwar et al., 2021). Counselors who demonstrate a high normative commitment may be more likely to interpret pay regulations in a more positive manner, and perceive pay systems as fair and acceptable, owing to their deeper sense of identification with organizational objectives and values (Oh et al., 2025).

### Emotional exhaustion

In addition to normative commitment and pay satisfaction, emotional exhaustion plays a central role in explaining how job demands influence employees’ attitudes and well-being. Emotional exhaustion acts as an essential mediating factor in this model. The JD-R model posits that emotional exhaustion is the main strain outcome of long-term exposure to high job demands when resources are insufficient. With counselors facing high levels of emotional exhaustion, the capacity of their psychological capacity to convert organizational attachment into favorable assessments of pay is compromised (Park et al., 2021). The emotional exhaustion decreases the tolerance for limited rewards (Zhang et al., 2025). For this reason, emotional exhaustion is regarded as a primary mechanism through which normative commitment affects pay satisfaction.

### Mediating role of emotional exhaustion

Building on the relationships discussed above, emotional exhaustion is expected to function as a mediating mechanism linking normative commitment and pay satisfaction. Within the Job Demands-Resources framework, organizational commitment can operate as a psychological resource that reduces the strain associated with demanding work conditions (Demerouti et al., 2001; Bakker and Demerouti, 2007). When counselors possess higher levels of normative commitment, they may experience lower levels of emotional exhaustion because their sense of moral obligation and institutional identification helps them cope with work pressures more effectively. Reduced emotional exhaustion, in turn, may enable counselors to evaluate organizational rewards more positively, including their satisfaction with pay (Wang et al., 2020; Park et al., 2021). Thus, emotional exhaustion is proposed to transmit part of the effect of normative commitment on pay satisfaction.

Overall, the proposed theoretical framework incorporates the JD-R model with the multidimensional conceptualization of organizational commitment to articulate how normative commitment drives emotional exhaustion and pay satisfaction among university counselors. By positioning emotional exhaustion as a mediating strain mechanism, the framework provides a coherent explanation of how organizational attitudes are transformed into compensation-related evaluations in emotionally demanding higher education contexts and lays the theoretical foundation for the proposed structural equation model tested in this study.

Based on this theoretical framework, the following hypotheses are proposed:

*H*1: Normative commitment has a negative impact on emotional exhaustion among university counselors.

*H*2: Normative commitment has a positive impact on pay satisfaction among university counselors.

*H*3: Emotional exhaustion has a negative impact on pay satisfaction among university counselors.

*H*4: Emotional exhaustion mediates the relationship between normative commitment and pay satisfaction among university counselors.

## Conceptual framework

**Figure 1:**
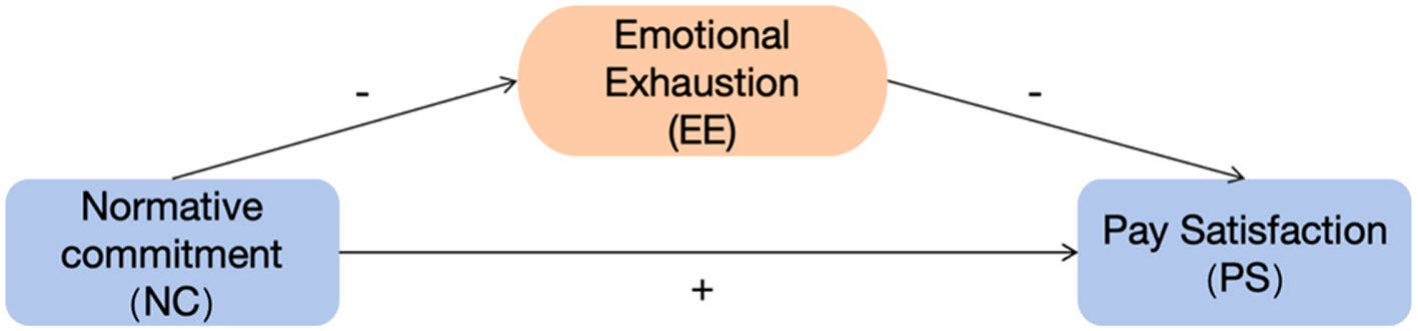
Conceptual model of the study.

Figure 1 summarizes the conceptual model in this study. The model shows the direct attitudinal pathway and the indirect strain-based pathway that links normative commitment to pay satisfaction within the context of higher education counseling.

## Methods

### Research design

This study employed a quantitative, cross-sectional design to explore the relationships among normative commitment, emotional exhaustion, and pay satisfaction among university counselors in China. The data were collected at a single time point to evaluate the associations between variables and to test the proposed mediation model.

### Subjects and data collection

The study population was university counselors from three Chinese universities. 300 university counselors were invited and recruited to participate in the study. Due to the screening for missing values and invalid responses, a total of 294 valid questionnaires were prepared for data analyses, and the effective response rate was 98%. The study employed convenience sampling, which is a non-probability sampling technique, to recruit participants. The participants were selected based on their accessibility and willingness to participate in the study. Mixed-mode (online and paper-based) data collection was used to improve the coverage and response quality. The questionnaire consisted of demographic information and work-related information, and standardized measures of normative commitment, emotional exhaustion, and pay satisfaction.

### Measures

The measurement of normative commitment was the normative commitment dimension of the Organizational Commitment Scale developed by Long et al. (2000). The subscale has 5 items scaled on a 5-point Likert scale from 1 (strongly disagree) to 5 (strongly agree). Higher numbers indicate stronger normative commitment.

The measurement of emotional exhaustion was the emotional exhaustion subscale of Chinese-translated version of Maslach Burnout Inventory (MBI) by Wang and Xu (2004). The subscale has 9 items and items were rated on a 7-point frequency scale from 1 (never) to 7 (always). Higher numbers indicate greater emotional exhaustion.

The dimension of pay satisfaction was assessed through the subscale of pay satisfaction of Job Satisfaction Questionnaire for University Teachers, developed by Yu (2007). The dimension of pay satisfaction had 4 items, and the items were rated on a 4-point Likert scale ranging from 1 (completely inconsistent) to 4 (completely consistent). Higher numbers indicate greater pay satisfaction.

### Data analysis

SPSS was used to conduct data analyses to examine the interrelationships among normative commitment, emotional exhaustion, and pay satisfaction. The descriptive statistics were performed first to obtain summaries of the central tendency and the variability of major variables. The independent samples t-tests and ANOVA were then conducted to test group differences based on selected demographic and work-related variables. The Pearson correlation analyses were then performed to assess the bivariate relationships, and to obtain the basic support for the suggested relations. To test the direct relationships between pairs of variables, a set of linear regression analyses was performed. Meanwhile, relevant demographic and occupational covariates were considered as control variables. The regression results were interpreted using standardized regression coefficients (*β*), t-values, *p*-values, and explained variance (R^2^). Multicollinearity was measured using the variance inflation factors (VIF). Furthermore, the mediating effect of emotional exhaustion was examined using a bootstrapping procedure with bias-corrected confidence intervals to construct an estimate of the indirect effect of normative commitment on pay satisfaction. The mediation was taken to be significant when the 95% bootstrap confidence interval failed to contain zero. Taken together, all these analyses provided an integrated assessment of the direct effects between study variables and the indirect pathway via emotional exhaustion.

### Reliability, validity, and normality

Before testing hypotheses, the reliability, validity, and normality of the measurement model were systematically evaluated. Table 1 indicates all loadings for normative commitment, emotional exhaustion, and pay satisfaction were greater than the acceptable cutoff point of 0.50, with most items loading higher than 0.70, suggesting satisfactory levels of indicator reliability. The internal consistency reliability was well established, with Cronbach’s alpha values ranging from 0.799 to 0.949 and composite reliability values ranging from 0.781 to 0.955, both of which were above the recommended cutoff-point of 0.700. Convergent validity was also confirmed, as average variance extracted (AVE) values ranged from 0.553 to 0.711, exceeding 0.50, and indicating each construct explained a greater share of variance in its indicators. Data normality was checks through skewness and kurtosis statistics, all of which were within acceptable ranges (skewness: −1.443 to 1.106; kurtosis: −0.812 to 3.440), exhibiting no substantial from normality.

**Table 1:** Internal consistency, convergent validity and normality results.

Construct	Item	Loadings	Skewness	Kurtosis	Α	CR	AVE
Normative commitment	NC1	0.724	0–0.629	−0.0174	0.798	0.804	0.553
	NC2	0.770	0–0.874	0.679			
	NC3	0.756	0–0.739	0.811			
	NC4	0.676	−1.037	0.660			
	NC5	0.788	−1.443	2.458			
Emotional exhaustion	EE1	0.724	0.693	−0.134	0.949	0.955	0.711
	EE2	0.770	0.385	−0.454			
	EE3	0.756	0.498	−0.0344			
	EE4	0.676	0.840	0.374			
	EE5	0.788	0.837	0.291			
	EE6	0.724	1.106	0.529			
	EE7	0.770	0.859	0.230			
	EE8	0.756	0.822	0.203			
	EE9	0.676	0.796	0.487			
Pay satisfaction	PS1	0.724	−0.523	0.257	0.769	0.781	0.590
	PS2	0.770	−0.737	0.319			
	PS3	0.756	−0.428	0.429			
	PS4	0.676	−0.731	1.004			

### Control variables

**Figure 2:**
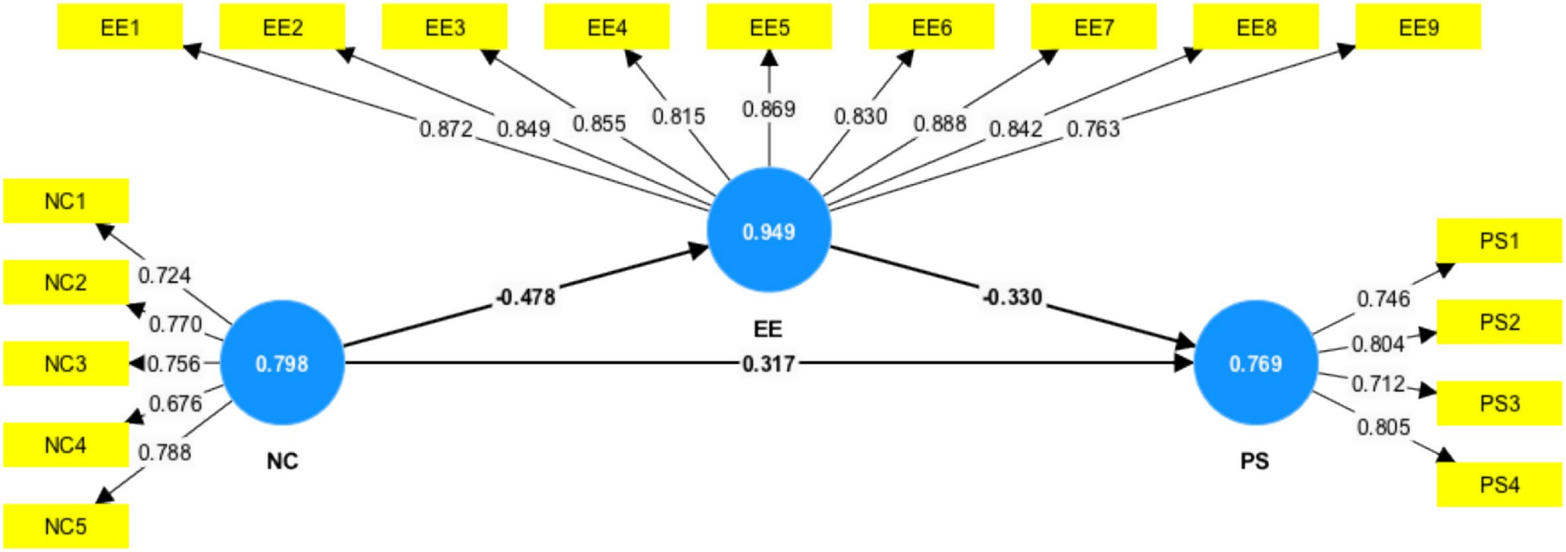
Measurement model.

To mitigate potential confounding, demographic variables (age, gender, marital status, and income level) and work-related variables (professional title, work experience) were considered as control variables in all regression and mediation analyses (Figure 2).

## Findings

The first set of results provides descriptive statistics of the variables, as detailed in Table 2. The counselors at the three universities had, in general, relatively high levels of normative commitment (M = 3.934, SD = 0.718) on a 5-point scale, suggesting that they had high moral commitment toward the institution. They felt moderately exhausted (M = 3.107, SD = 1.259) on a 7-point scale, suggesting that counselors undergo a noticeable level of emotional exhaustion, but it is not extreme. Similarly, pay satisfaction was on a moderate to high level (M = 3.100, SD = 0.550) on a 4-point scale. This indicates that counselors, in general, evaluate their compensation positively. The measured standard deviation suggests sufficient variability across all the variables, thereby making them appropriate for future correlation, regression, and mediation analyses.

**Table 2:** Descriptive statistics.

Variable	Maximum	Mean	Standard deviation
Normative commitment	5	3.934	0.718
Emotional exhaustion	7	3.107	1.259
Pay satisfaction	4	3.100	0.550

Table 3 reports group differences in the variables across demographic and work-related characteristics. Overall, normative commitment did not differ significantly by age, gender, marital status, work experience, or professional title, but it varied significantly by income level (*F* = 3.639, *p* < 0.01), indicating that counselors’ sense of moral obligation toward the institution differs across income groups. The same was observed for emotional exhaustion, which did not differ significantly by age, gender, marital status, or professional title; however, it did differ by work experience (*F* = 3.225, *p* < 0.05) and income (*F* = 2.946, *p* < 0.05), implying both factors may be associated with counselors’ emotional exhaustion. Pay satisfaction also demonstrated significant differences by age (*F* = 2.475, *p* < 0.05) and, more strongly, by income level (*F* = 5.630, *p* < 0.001), but non-significant differences according to gender, marital status, work experience, and professional title. The findings indicate income as the most uniform distinguishing factor across the three variables whereas age and work experience are more selective in their association (age with pay satisfaction; work experience with emotional exhaustion). Regarding these findings, demographic and work-related variables, especially income, were used as control variables in further analyses because they were treated as potential confounds.

**Table 3:** Differences in demographic variables.

Variable	Age F	Gender T	marital status T	Work experience F	Professional title F	Income F
Normative commitment	0.778	0.057	0.222	0.586	1.128	3.639**
Emotional exhaustion	1.663	0.736	0.346	3.225*	1.642	2.946*
Pay satisfaction	2.475*	0.465	0.183	1.418	0.044	5.630***

*p < 0.05, **p < 0.01, ***p < 0.001. This notation applies to all statistical results reported in the study.

Table 4 reports the Pearson correlation coefficients among normative commitment, emotional exhaustion, and pay satisfaction. Results indicate that normative commitment significantly and negatively correlates with emotional exhaustion (*r* = −0.471, *p* < 0.01), meaning counselors reporting stronger feelings of moral obligation and organizational loyalty experience fewer depressive feelings of emotional exhaustion. Moreover, normative commitment correlates positively with pay satisfaction (*r* = 0.465, *p* < 0.01), meaning higher normative commitment is a characteristic of counselors who tend to attribute their pay and benefits to positive evaluations. Finally, emotional exhaustion significantly and negatively correlates with pay satisfaction (*r* = −0.473, *p* < 0.01), meaning emotionally exhausted counselors tend to judge their pay levels lower. Correlation results show meaningful associations among the variables and justify further investigation of the direct and indirect mechanisms among them.

**Table 4:** Pearson correlation coefficients among normative commitment, emotional exhaustion, and pay satisfaction.

Variable	1	2	3
1 Normative commitment	--		
2 Emotional exhaustion	−0.471**	--	
3 Pay satisfaction	0.465**	−0.473**	--

*p < 0.05, **p < 0.01, ***p < 0.001.

The direct effects of study variables in regression are displayed in Table 5. Following H1, a significant negative effect was observed between NC and EE (*β* = −0.771, SE = 0.092, *t* = −8.419, *p* < 0.001; standardized Beta = −0.440), implying that counselors with higher moral duty and institutional pride had a lower level of EE. The model accounted for a significant proportion of variance in EE (R^2^ = 0.285; adjusted R^2^ = 0.268), and collinearity did not arise (VIF = 1.091).

**Table 5:** Direct effect results.

Construct	Hypothesis	β	SE	t-value	Beta	*p*-value	R^2^	Adjusted R^2^	F	VIF	Decision
NC → EE	H1	−0.771	0.092	−8.419	−0.440	<0.001	0.285	0.268	16.288	1.091	Accepted
NC → PS	H2	0.324	0.041	7.945	0.423	<0.001	0.257	0.239	14.146	1.091	Accepted
EE → PS	H3	−0.196	0.023	−8.379	−0.448	<0.001	0.272	0.254	15.262	1.121	Accepted

NC, Normative Commitment; EE, Emotional Exhaustion; PS, Pay Satisfaction. This notation applies to all results reported in the study.

In support of H2, it was also noticed that NC had a positive effect on PS (*β* = 0.324, SE = 0.041, *t* = 7.945, *p* < 0.001; standardized Beta = 0.423), implying that a sense of duty accompanies a favorable evaluation of compensation (R^2^ = 0.257; adjusted R^2^ = 0.239; VIF = 1.091).

In support of H3, EE also exhibited a significant negative impact on PS (*β* = −0.196, SE = 0.023, *t* = −8.379, *p* < 0.001; standardized Beta = −0.448), indicating that emotionally exhausted counselors are less satisfied with their compensation. This model also exhibited acceptable collinearity (R^2^ = 0.272; adjusted R^2^ = 0.254; VIF = 1.121). The direction, magnitude, and significance of the coefficients give strong support to the hypothesized direct associations and provide basis for the further mediation test of EE in the NC-PS relation (Table 6).

**Table 6:** Mediation analysis of emotional exhaustion in the relationship between normative commitment and pay satisfaction.

	Effect	Effect ratio	Bootstrap SE	Bootstrap 95%	
				Boot LLCI	Boot ULCI
NC → EE → PS					
Effect	0.324	100.00%	0.041	0.244	0.404
Direct effect	0.216	66.718%	0.043	0.131	0.302
Indirect effect	0.108	33.282%	0.029	0.057	0.169

The overall effect of NC on PS was 0.3242 (SE = 0.0408, 95% CI [0.2438, 0.4044]), indicating a significant relationship between NC and PS independently. The direct impact of NC on PS after controlling for EE was 0.2163, SE = 0.0433, 95%CI [0.1310, 0.3016], accounting for 66.72% of the observed effect, indicating that the contribution of NC to PS was independent of EE. Furthermore, the indirect effect of NC on PS via EE was 0.1079 SE = 0.0290, 95%CI [0.0567, 0.1692], representing 33.28% of the total effect. There has been no zero in the 95% Bias Corrected bootstrap confidence interval for the indirect effect, implying that the mediation effect is statistically significant. These results align with an empirical confirmation of a partial mediation model where EE plays a role in the relationship between NC and PS, partially mediating the effects of NC on PS. This information sheds light on the dual pathway, both direct and indirect, through which NC can contribute to PS, and the important intermediary role that EE plays in that process.

### Ethical issues

This study was performed in line with the ethical standards for research involving humans. The participants provided informed consent after being informed of the objective and procedures of the study. The data was used anonymously, and no personal identifying information was shared to a third party. The participants were also informed of their right to withdraw from the study anytime without any consequences. There was no known psychological risk imposed on the participants for being involved in this study. Data were stored securely and used only for research purposes.

## Discussion

In line with our theorizing, we accepted H1 suggesting a significant negative relationship between NC and EE (*β* = −0.771, SE = 0.092, t = 8.419, standardized *β* = −0.440, *p* < 0.001), with the model accounting for a meaningful amount of variance in EE (R^2^ = 0.285; *F* = 16.288; VIF = 1.091). Also, we accepted H2, indicating that NC is positively and directly related to PS (*β* = 0.324, SE = 0.041, *t* = 7.945, standardized *β* = 0.423, *p* < 0.001; R^2^ = 0.257; *F* = 14.146; VIF = 1.091). We accepted H3, showing that EE is significantly and negatively associated with PS (*β* = −0.196, SE = 0.023, *t* = 8.379, standardized *β* = −0.448, *p* < 0.001; R^2^ = 0.272; *F* = 15.262; VIF = 1.121). This pattern indicates that counselors’ sense of moral obligation toward the institution relates to more favorable compensation evaluations beyond the intervening role of strain. This finding is theoretically meaningful in counselor work contexts characterized by chronic emotional demands: NC can be conceptualized as a job resource that helps sustain meaning, duty-based motivation, and persistence, thereby buffering the depletion of EE. Finally, H4 was supported. Bootstrap findings revealed a strong indirect impact of NC on PS via EE (*β* = 0.1079, SE = 0.0290, 95% CI [0.0567, 0.1692]). Considering that the direct effect was statistically significant despite the fact that the indirect channel was still significant, we believe the best description for mediation is partial mediation such that the mediated effect accounted for 33.28% of the overall effect (direct = 66.72%).

Together, the current findings support direct and indirect NC influencing PS through diminishing EE. The overall findings inform literature in three directions. First, in terms of Conservation of Resources, EE is a state of resource depletion. Our results suggest that NC serves as a resource that protects counselors from running out of emotional energy which, if not conserved elsewhere, could cascade into negative evaluatively appraising organizational returns (Hobfoll, 1989, 2001). Second, in line with the JD-R model, counselors encounter high demands of emotion and role; the current evidence indicates that attachment based on NC can serve as a resource that buffers burnout processes and can maintain positive work attitudes (Bakker and Demerouti, 2007; Demerouti et al., 2001). Third, the observed EE → PS association can be interpreted in addition through Effort–Reward Imbalance logic: increases in exhaustion can trigger greater feelings that effort expended is not sufficiently returned, which can negatively impact compensation appraisal (Siegrist, 1996; van Vegchel et al., 2005).

The findings of this study are generally consistent with previous research examining the relationships among organizational commitment, emotional exhaustion, and job-related attitudes. Earlier studies have shown that organizational commitment can influence employees’ perceptions of their work environment and compensation systems, while emotional exhaustion often functions as an important psychological mechanism linking workplace demands with job satisfaction outcomes (Wang et al., 2020; Park et al., 2021). Recent studies have similarly emphasized that emotional exhaustion plays a crucial mediating role in shaping employees’ attitudes toward their work and organizational rewards, particularly in emotionally demanding professions such as education and counseling (Lesener et al., 2019; Kim et al., 2025). The present findings extend this line of research by demonstrating that normative commitment not only directly influences pay satisfaction but also indirectly affects it through emotional exhaustion among university counselors. This result contributes to the existing literature by highlighting the importance of emotional well-being in shaping employees’ perceptions of organizational rewards in higher education contexts.

### Implications

The findings produce multiple actionable implications for higher education management and human resource practice. First, the positive association between NC and PS suggests that universities should reinforce counselors’ value-based attachment to the institution through support-oriented commitment-building practices. Thus, institutions might invest in structured professional development, transparent career pathways, and recognition systems that convey institutional reciprocity and legitimacy, bolstering counselors’ perceived alignment with institutional goals and acceptance of organizational obligations (Hobfoll, 2001).

Second, the mediating role of EE underscores that improving PS requires burnout prevention as a complementary lever, not merely a compensation issue. A growing body of evidence has found a stronger psychosocial safety climate to be a buffer for strain processes and EE in settings characterized by high job requirements (Loh et al., 2018). In a real-world setting, universities should integrate a multi-modal burnout intervention that includes (a) workload and role regulation (e.g., caseload limits, peak-period staffing, explicit performance expectations), (b) resource optimization (e.g., high-quality supervision, peer consultation, recovery time), and (c) available psychological help.

Last, as income-related variations were also noted in EE and PS, universities should explicitly address the effort-reward imbalance embedded within counseling work. According to effort-reward imbalance theory, effort-reward imbalance can amplify strain, which can deteriorate satisfaction assessments-including pay evaluations (Siegrist, 1996; van Vegchel et al., 2005). Empirical studies, particularly in challenging human service settings, have also revealed that effort-reward imbalance related processes are strongly linked to EE (Liang et al., 2023). Thus, compensation reform should focus on pay fairness, transparency, and reward diversity, including a combination of salary progress and non-monetary compensation (e.g., protected time for training, research/innovation grants, licensure support, and acknowledgement for high emotional effort). PS has also been found to correlate with more comprehensive quality-of-life measurements, implying that compensation schemes might have tangible effects on well-being outcomes beyond immediate financial assessment (Ma et al., 2025).

### Limitations

Although it brings some contributions, some restrictive factors necessitate caution. Firstly, the cross-sectional design avoids causal inference; a longitudinal or experimental study is required to establish ordering over time for NC, EE, and PS. Second, the sample consisted of individual participants from three universities, which could limit the generalizability; if possible, future research should aim to replicate the model on larger samples and across a larger range of institutional types and regions. Third, self-report data were used exclusively, creating potential for common method bias; using multi-source data designs are encouraged. Finally, a limited number of variables were presented in the model; including additional job demands and resources (e.g., workload, autonomy, supervisory support), organizational context (e.g., climate, culture), and individual factors (e.g., coping, resilience) will offer a more complete explanation of counselors’ well-being and attitudes toward compensation.

## Data Availability

The raw data supporting the conclusions of this article will be made available by the authors, without undue reservation.
