# Correction: The mediating role of emotional exhaustion in the relationship between normative commitment and pay satisfaction among university counselors

**DOI:** 10.3389/fpsyg.2026.1875192

**Published:** 2026-05-26

**Authors:** Zeng Cheng, Esayas Teshome Taddese, Bereket Merkine Gebresilase, Mitiku Tasisa Dinsa, Kelemu Zelalem Berhanu, Melaku Takele Abate, A. Shorouk

**Affiliations:** 1 Faculty of Education and Liberal Arts, INTI International University, Nilai, Malaysia; 2 College of Physics and Electronic Information Engineering, Minjiang University, Fuzhou, China; 3 School of Nursing, Shandong Xiehe University, Jinan, China; 4 Department of English Language and Literature, College of Social Science and Humanities, Wolkite University, Welkite, Ethiopia; 5 Department of Education Leadership and Management, University of Johannesburg, Johannesburg, South Africa; 6 Jimma College of Teachers' Education, Jimma, Ethiopia; 7 Department of Education, College of Humanities and Science, Ajman University, Ajman, United Arab Emirates

**Keywords:** education, educational governance, emotional exhaustion, normative commitment, pay satisfaction

In the published article, Author Zeng Cheng was erroneously assigned to affiliation “School of Teacher Education, Minjiang University, Fuzhou, China” The correct affiliation is “College of Physics and Electronic Information Engineering, Minjiang University, Fuzhou, China”. Esayas Teshome Taddese was erroneously assigned to affiliation “School of Teacher Education, Minjiang University, Fuzhou, China”. The correct affiliation for Esayas Teshome Taddese is “Faculty of Education and Liberal Arts, INTI International University, Nilai, Malaysia”. Bereket Merkine Gebresilase was erroneously assigned to affiliation “School of Psychology, Shandong Xiehe University, Jinan, China”. The correct affiliation for Bereket Merkine Gebresilase is “School of Nursing, Shandong Xiehe University, Jinan, China.”

The original version of this article has been updated.

